# Genetic sub-structuring of Croatian island populations in the Southeastern European context: a meta-analysis

**DOI:** 10.3325/cmj.2022.63.231

**Published:** 2022-06

**Authors:** Natalija Novokmet, Ana Galov, Vedrana Škaro, Petar Projić, Jelena Šarac, Dubravka Havaš Auguštin, Pavao Rudan, Dragan Primorac, Damir Marjanović

**Affiliations:** 1 Institute for Anthropological Research, Zagreb, Croatia; 2 Department of Animal Physiology, Faculty of Science, Zagreb, Croatia; 3 DNA Laboratory, Genos Ltd., Zagreb, Croatia; 4 Laboratory for Molecular Anthropology, Center for Applied Bioanthropology, Institute for Anthropological Research, Zagreb, Croatia; 5 Scientific Council for Anthropological Research, Croatian Academy of Sciences and Arts, Zagreb, Croatia; 6 St. Catherine Hospital, Zagreb, Croatia; 7 School of Medicine, University of Split, Split, Croatia; 8 University Department of Forensic Sciences, University of Split, Split, Croatia; 9 School of Medicine Rijeka, University of Rijeka, Rijeka, Croatia; 10 Faculty of Medicine, University of Osijek, Osijek, Croatia; 11 Faculty of Dental Medicine and Health, University of Osijek, Osijek, Croatia; 12 Eberly College of Science, Penn State University, University Park, PA, USA; 13 Henry C. Lee College of Criminal Justice and Forensic Sciences, University of New Haven, West Haven, CT, USA; 14 Medical School REGIOMED, Coburg, Germany; 15 The National Forensic Sciences University, Gandhinagar, Gujarat, India; 16 Department of Genetics and Bioengineering, International Burch University, Sarajevo, Bosnia and Herzegovina

## Abstract

**Aim:**

To use the method of meta-analysis to assess the influence of island population isolation on the sub-structuring of the Croatian population, as well as the influence of regional population groups on the sub-structuring of the Southeastern European population with regard to basic population genetic statistical parameters calculated by using STR locus analysis.

**Methods:**

Bio-statistical analyses were performed for 2877 unrelated participants of both sexes from Southeastern Europe. Nine autosomal STR loci (D3S1358, vWA, FGA, TH01, TPOX, CSF1PO, D5S818, D13S317, and D7S82) were analyzed by using standard F-statistics and population structure analysis (Structure software).

**Results:**

Genetic differentiation of Croatian subpopulations assessed with the F_ST_ method was higher at the level of the Croatian population (0.005) than at the level of Southeastern Europe (0.002). The island of Vis showed the most pronounced separation in the Croatian population, and Albanians from Kosovo in the population of Southeast Europe, followed by Croatia, Bosnia and Herzegovina, and Hungary.

**Conclusion:**

The higher structure of Croatian subpopulations in relation to Southeastern Europe suggest a certain degree of genetic isolation, most likely due to the influence of endogamy within rural island populations.

The island populations of the eastern Adriatic have been the subject of multidisciplinary anthropological research for almost 50 years, starting with the pioneering work of Rudan et al in 1972 (1). A number of specific features of these rural populations has been revealed, which make them exceptional models for studying ethno-cultural, historical, migratory, and demographic characteristics of this region. More specifically, evolutionary forces (bottleneck effect and genetic drift) increase genome homogeneity within the genetic structure of such island isolates by eliminating certain genetic traits in favor of others and increasing the likelihood of finding low-impact alleles (2,3). The reduced genetic and environmental diversity makes genetically isolated populations suitable for the study of different complex and rare Mendelian hereditary diseases, since the combined action of genetic drift, inbreeding, and founder effect increases the prevalence of such diseases when compared with the general population.

Southeastern Europe was one of Europe’s glacial refugia during the ice age, and the origin of postglacial resettlement of Europe in the Paleolithic and Neolithic. Due to this specific role and its position at the crossroads of migrations to and from Europe, this area was extensively investigated in the field of population genetics (4-7). Different genetic markers have been used to investigate the genetic landscape of Europe and determine the patterns of population sub-structuring at the regional and continental level (8). As a part of the comprehensive anthropological research on the population structure of Croatian island isolates, microsatellite DNA from different subpopulations has been previously analyzed to determine genetic diversity, population structure, and the degree of isolation of island populations (9). Similar studies were also conducted on a representative sample of the general Croatian population and other isolated populations from Southeastern Europe (10-12).

This study represents a continuation of previous anthropogenetic research (6,13-16). We used statistical and analytical methods of meta-analysis to synthesize data from previously conducted, mutually independent studies of island and continental populations of Croatia and Southeastern Europe (Figure 1) based on analyses of autosomal STR markers, and data analyzed in this study for the first time.

**Figure 1:**
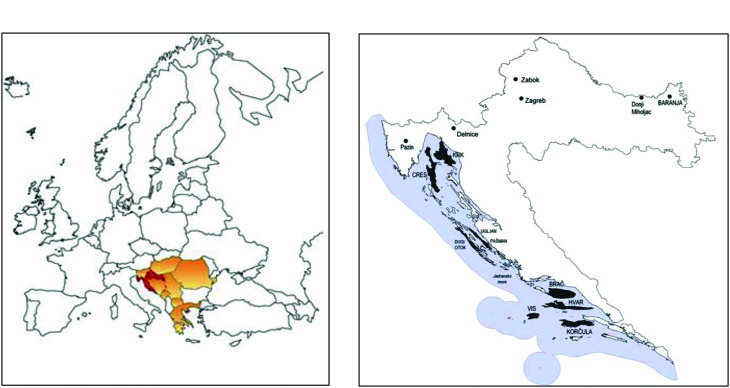
Division of the sample into two hierarchical groups (left) and geographical position of the investigated Croatian subpopulations (right). NDI – North Dalmatian islands.

The aim of this study was to determine the genetic characteristics of populations from Southeastern Europe, with special reference to Croatian island populations, and to investigate the effect of specific intrapopulation genetic structure on interpopulation relationships. Namely, a specific aim was to investigate the influence of island population isolation on the sub-structuring of the Croatian population, and the influence of regional population groups on the sub-structuring of Southeastern Europe with regard to basic population genetic statistical parameters calculated by using STR locus analysis.

## Material and methods

### Sample

The samples used were the same as described in a previous article by our research group (17). Certain analyses of autosomal STR markers were conducted for the first time in this study and some are from previous research performed by various authors (9,14-16,18-28). This article integrates all these studies using the statistical and analytical method of meta-analysis, as defined by Rosenbald (29).

### STR marker analysis

STR marker analysis was performed by salting out method (30) as described in a previous article by our research group (17).

### Bio-statistical analyses

Since data for 2877 samples were unavailable for all the loci included in the AmpFLSTR Identifiler PCR Amplification Kit, to allow data comparison, bio-statistical analyses were conducted for nine autosomal STR loci (D3S1358, vWA, FGA, TH01, TPOX, CSF1PO, D5S818, D13S317, D7S82).

The standard F-statistics (31), which describes the level of inbreeding within a subpopulation (F_IS_), between subpopulations (F_ST_), and within the total population (F_IT_), was used as a measure of correlation between alleles. Clustering was performed with the Ward hierarchical method (32,33) and the results were presented as dendrograms created with Statistica 9 (StatSoft, TIBCO Software, Dell, Round Rock, TX, USA).

To determine whether there are significant differences in allelic frequencies, ie, population structure in the entire population of isolated island subpopulations in Croatia and among the populations of Southeast Europe, a population structure analysis was performed (34) by using the program Structure 2.3.3 (Stanford University, Stanford, CA, USA). The limitation of low levels of population differentiation (FST<0.02) was overcome by using the sampling location parameter (35). The “LocPrior” option of program settings, which enables the determination of the structure at lower levels of separation was used (36). Tests K from 1 to 10 were performed, and each run was repeated 10 times. To determine the most reliable K value, ΔK was calculated based on the rate of change lnP (D) between individual K values. In order to determine ΔK, the results of all analyses were processed in Structure Harvester v. 0.6 (37,38).

The analysis conducted at the level of the Croatian population included a sample of all island subpopulations (n = 733), excluding the mainland population. The analysis conducted at the level of Southeastern Europe included a sample of Southeastern European populations (n = 1805). The Croatian sample analyzed in the broader Southeastern European context was reduced from n = 1230 to n = 158 to mirror the portions of these populations in the actual sample of Croatia and to avoid sample bias (39).

## Results

### Genetic differentiation of Croatian subpopulations

The total genetic differentiation coefficients (F_ST_) for the compared population pairs (Table 1) and for each analyzed locus (Table 2) were estimated to assess the genetic distances between the analyzed Croatian subpopulations. Most of the analyzed pairs showed a relatively small but significant level of genetic differentiation. The only exceptions were two analyzed pairs: mainland-island of Brač and island of Krk-North Dalmatian islands (NDI), with no significant difference at any locus. The lowest degree of total genetic differentiation was found between the mainland and the island of Brač (0.06%), with no significant difference at any locus. The highest degree of total genetic differentiation was found between Hvar and Vis (F_ST_ = 1.6%), followed by Cres-Vis, and Korčula-Vis, with the same degree of F_ST_ = 1.4%. Vis showed the highest values of genetic differentiation compared with all other analyzed populations. The observed values for this island ranged from 0.7%, when compared with the mainland and NDI to 1.6%, when compared with Hvar.

**Table 1:** Total genetic differentiation coefficient (F_ST_) between individual pairs of Croatian (n = 1230) subpopulations. Values in bold indicate significant results: Fst values (below the diagonal) and p- values (above the diagonal)

	Mainland* n = 497	Islands n = 733						
		Krk n = 137	Cres n = 122	NDI^†^ n = 82	Brač n = 96	Hvar n = 103	Korčula n = 95	Vis n = 98
Mainland*		**0.00000**	**0.00000**	**0.02703**	0.19820	**0.00000**	**0.00000**	**0.00000**
Krk	**0.00232**		**0.00000**	0.09009	**0.00901**	**0.00901**	**0.00000**	**0.00000**
Cres	**0.00669**	**0.00722**		**0.00000**	**0.00000**	**0.00000**	**0.00000**	**0.00000**
NDI^†^	**0.00136**	0.00145	**0.00518**		**0.01802**	**0.00000**	**0.00901**	**0.00000**
Brač	0.00063	**0.00240**	**0.00493**	**0.00401**		**0.00000**	**0.00000**	**0.00000**
Hvar	**0.00460**	**0.00349**	**0.01309**	**0.00713**	**0.00433**		**0.00000**	**0.00000**
Korčula	**0.00391**	**0.00457**	**0.01040**	**0.00413**	**0.00501**	**0.00502**		**0.00000**
Vis	**0.00690**	**0.00827**	**0.01364**	**0.00692**	**0.00777**	**0.01622**	**0.01360**	

*Mainland: Baranja and mainland Croatia (Zagreb, Pazin, Delnice, Zabok, and Donji Miholjac).

†North Dalmatian islands: Ugljan, Pašman, Dugi otok.

**Table 2:** Coefficient of genetic differentiation (F_ST_ – *P* values) for each individual locus (AMOVA) in the analyzed Croatian subpopulations (mainland n = 497, Krk n = 137, Cres n = 122, North Dalmatian islands [NDI, Ugljan, Pašman, Dugi otok] n = 82, Brač n = 96, Hvar n = 103, Korčula n = 95, Vis n = 98). Values in bold indicate significant results

Locus	Mainland* vs island Krk	Mainland* vs island Cres	Mainland* vs NDI*	Mainland* vs island Brač	Mainland* vs island Hvar	Mainland* vs island Korčula
**D3S1358**	**0.048**	**0.002**	0.168	0.673	0.072	**0.009**
**VWA**	0.635	0.369	0.215	0.239	0.089	0.808
**FGA**	0.357	0.108	**0.002**	0.172	0.132	0.631
**TH01**	0.214	**0.031**	0.300	0.298	0.367	0.095
**TPOX**	**0.048**	0.219	0.542	0.885	**0.008**	**0.020**
**CSF1PO**	0.097	**0.000**	0.263	0.094	**0.012**	0.587
**D5S818**	0.320	**0.023**	0.316	0.076	**0.029**	0.321
**D13S317**	0.174	**0.001**	0.541	0.305	**0.006**	0.115
**D7S820**	**0.000**	**0.003**	0.508	0.641	0.229	**0.000**
**Locus**	Mainland* vs island Vis	Island Krk vs island Cres	Island Krk vs NDI	Island Krk vs island Brač	Island Krk vs island Hvar	Island Krk vs island Korčula
**D3S1358**	**0.005**	**0.022**	0.570	0.095	0.780	0.153
**VWA**	0.061	0.721	0.062	0.140	0.189	0.897
**FGA**	**0.000**	0.127	0.179	0.107	**0.012**	0.108
**TH01**	0.576	**0.030**	0.215	0.368	0.081	**0.008**
**TPOX**	**0.007**	**0.012**	0.153	0.242	0.121	0.057
**CSF1PO**	**0.005**	**0.017**	0.399	0.927	0.226	0.129
**D5S818**	**0.039**	**0.011**	0.226	0.160	0.505	0.509
**D13S317**	**0.029**	0.329	0.690	0.231	**0.023**	0.646
**D7S820**	**0.009**	**0.000**	0.342	**0.040**	**0.038**	**0.000**
**Locus**	Island Krk vs island Vis	Island Cres vs NDI	Island Cres vs island Brač	Island Cres vs island Hvar	Island Cres vs island Korčula	Island Cres vs island Vis
**D3S1358**	**0.000**	**0.021**	**0.016**	**0.021**	**0.001**	**0.000**
**VWA**	0.129	0.104	0.282	0.356	0.432	0.316
**FGA**	**0.041**	0.078	**0.004**	0.079	0.081	**0.000**
**TH01**	0.148	**0.011**	0.342	0.583	0.209	**0.034**
**TPOX**	**0.000**	0.631	0.208	**0.003**	**0.012**	0.124
**CSF1PO**	0.517	0.154	**0.006**	**0.000**	**0.001**	0.065
**D5S818**	**0.026**	0.165	0.228	**0.012**	0.075	**0.019**
**D13S317**	**0.003**	0.184	0.083	**0.000**	0.158	**0.004**
**D7S820**	0.478	0.105	0.294	**0.000**	**0.002**	**0.001**
**Locus**	NDI vs island Brač	NDI vs island Hvar	NDI vs island Korčula	NDI vs island Vis	Island Brač vs island Hvar	Island Brač vs island Korčula
**D3S1358**	0.062	0.352	0.308	**0.004**	0.089	**0.012**
**VWA**	0.304	**0.018**	0.233	0.071	0.332	0.133
**FGA**	**0.001**	**0.010**	**0.020**	**0.006**	**0.010**	0.315
**TH01**	0.050	0.086	**0.043**	0.457	0.524	0.128
**TPOX**	0.450	**0.037**	0.065	**0.031**	**0.048**	0.131
**CSF1PO**	0.239	**0.047**	0.148	0.231	0.483	0.214
**D5S818**	0.163	0.085	0.733	0.245	0.311	0.420
**D13S317**	0.724	0.050	0.860	**0.033**	**0.009**	0.265
**D7S820**	0.768	0.222	**0.036**	0.153	0.115	**0.010**
**Locus**	Island Brač vs island Vis	Island Hvar vs island Korčula	Island Hvar vs island Vis	Island Korčula vs island Vis
**D3S1358**	**0.018**	0.567	**0.003**	**0.006**
**VWA**	0.275	0.190	**0.039**	**0.042**
**FGA**	**0.002**	0.707	**0.000**	**0.000**
**TH01**	0.165	0.697	0.267	0.160
**TPOX**	**0.044**	0.152	**0.000**	**0.000**
**CSF1PO**	0.485	0.098	0.219	**0.019**
**D5S818**	**0.002**	0.420	**0.004**	**0.040**
**D13S317**	0.110	**0.019**	**0.000**	**0.015**
**D7S820**	**0.017**	**0.000**	0.064	**0.012**

*Mainland: Baranja and mainland Croatia.

**Figure 2:**
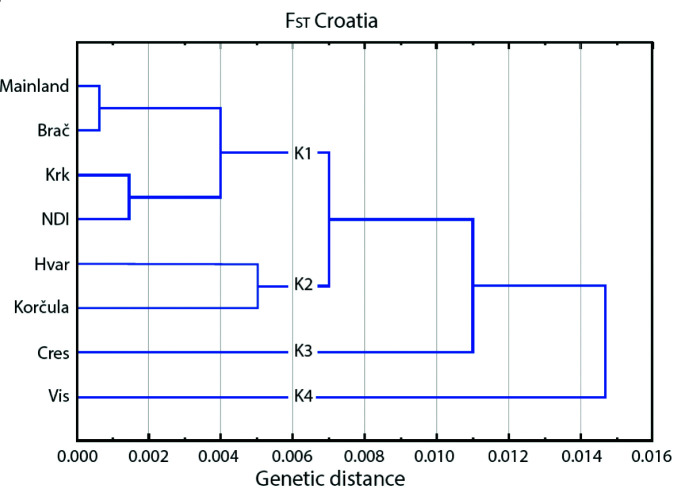
Clustering dendrogram for Croatian subpopulations obtained by Ward’s method (Ward et al. 1963). NDI – North Dalmatian islands.

The grouping results for Croatian subpopulations based on F_ST_ genetic distances, obtained by the Ward method, are presented as a dendrogram (Figure 2). Four main clusters are visible. The first one includes the populations of the mainland and the islands of Brač, Krk and NDI. The second cluster includes the islands of Hvar and Korčula. The islands of Cres and Vis can be considered the third and the fourth cluster, respectively. These two islands also have the smallest population, which affected the reduced genetic diversity within the islands, and the greater distance from other analyzed subpopulations. The population of Vis was distant from all the analyzed populations, and the most distant from Hvar.

### Genetic differentiation of the populations of Southeastern Europe

Total values of F_ST_ (Table 3) and the values of the genetic differentiation coefficient at each individual locus (Table 4) were estimated for each pair of populations to determine the kinship level. In contrast to the Croatian subpopulations for which significant levels of genetic differentiation were found in 93% of the analyzed pairs, the populations of Southeast Europe showed a significant level in only 56% of population pairs. The lowest degree of genetic differentiation was present between the populations of Serbia and Romania (0.00013), and Serbia and Montenegro (0.00023). Significant differences between these populations were not found at any locus. On the other hand, the highest degree of genetic differentiation was found between the populations of Hungary and Albanians from Kosovo (0.00737), where significant differences were observed at four loci (TH01, TPOX, CSF1PO, D7S820). Additionally, the population of Albanians from Kosovo was significantly different from all other populations except from North Macedonian population and had the highest values of genetic differentiation compared with all other populations. The established values for this population ranged from 0.2% when compared with the population of Romania to 0.7% when compared with the population of Hungary. For Bosnia and Herzegovina, a significant difference was found only with the population of Albanians from Kosovo (0.6%) and the population of Greece (0.2%).

**Table 3:** Total genetic differentiation coefficient (F_ST_) between individual pairs of Southeast European populations (n = 2877). Values in bold indicate significant results: Fst values (below the diagonal) and p- values (above the diagonal)

	Montenegro n = 101	Serbia n = 356	Croatia n = 1230	North Macedonia n = 100	Hungary n = 223	Bosnia and Herzegovina n = 100	Romania n = 222	Kosovo (Albanians) n = 137	Greece n = 297	Slovenia n = 111
Montenegro		0.30631	**0.00000**	0.46847	0.18018	0.18018	0.88288	**0.00000**	0.19820	**0.00901**
Serbia	0.00023		**0.00901**	0.16216	**0.03604**	0.54054	0.37838	**0.00000**	0.05405	**0.02703**
Croatia	**0.00220**	**0.00099**		**0.01802**	**0.04505**	0.56757	**0.00000**	**0.00000**	**0.00000**	0.20721
North Macedonia	-0.00002	0.00086	**0.00147**		**0.02703**	0.10811	0.75676	0.09910	0.37838	0.08108
Hungary	0.00073	**0.00107**	**0.00062**	**0.00221**		0.65766	**0.01802**	**0.00000**	**0.02703**	**0.01802**
Bosnia and Herzegovina	0.00095	-0.00019	-0.00004	0.00126	-0.00050		0.21622	**0.00000**	**0.00901**	0.51351
Romania	-0.00084	0.00013	**0.00119**	-0.00093	**0.00150**	0.00055		**0.00000**	**0.03604**	**0.00901**
Kosovo (Albanians)	**0.00573**	**0.00362**	**0.00569**	0.00153	**0.00737**	**0.00602**	**0.00240**		**0.00000**	**0.00000**
Greece	0.00060	0.00083	**0.00255**	-0.00007	**0.00096**	**0.00180**	**0.00090**	**0.00401**		**0.00000**
Slovenia	**0.00287**	**0.00206**	0.00064	0.00118	**0.00166**	-0.00023	**0.00217**	**0.00625**	**0.00340**	

**Table 4:** Coefficient of genetic differentiation (F_ST_-*P* values) for each individual locus (AMOVA) in the compared pairs of populations of Southeast Europe (Montenegro n = 101, Serbia n = 356, Croatia n = 1230, North Macedonia n = 100, Hungary n = 223, Bosnia and Herzegovina n = 100, Romania n = 222, Kosovo n = 137). Values in bold indicate significant results

Locus	Montenegro vs Serbia	Montenegro vs Croatia	Montenegro vs North Macedonia	Montenegro vs Hungary	Montenegro vs Bosnia and Herzegovina	Montenegro vs Romania	Montenegro vs Kosovo (Albanians)
**D3S1358**	0.929	0.545	0.647	0.428	0.983	0.861	0.078
**VWA**	0.303	0.193	0.154	0.234	0.427	0.329	0.410
**FGA**	0.067	0.062	0.214	**0.046**	0.051	0.549	0.020
**TH01**	0.382	**0.042**	0.881	0.116	0.110	0.952	0.606
**TPOX**	0.205	0.326	0.159	0.566	0.613	0.148	0.005
**CSF1PO**	0.848	0.672	0.541	0.848	0.345	0.895	0.004
**D5S818**	0.198	0.058	0.557	0.283	0.072	0.451	0.365
**D13S317**	0.571	0.137	0.326	0.610	0.862	0.288	0.131
**D7S820**	0.341	**0.014**	0.550	0.233	0.278	0.769	0.018
							
**Locus**	Montenegro vs Greece	Montenegro vs Slovenia	Serbia vs Croatia	Serbia vs North Macedonia	Serbia vs Hungary	Serbia vs Bosnia and Herzegovina	Serbia vs Romania
							
**D3S1358**	0.582	0.317	0.149	0.413	0.549	0.986	0.463
**VWA**	0.114	0.117	0.595	0.439	0.552	0.929	0.753
**FGA**	0.082	0.077	0.081	0.337	0.117	0.482	0.389
**TH01**	0.933	0.063	**0.039**	0.159	0.107	0.098	0.259
**TPOX**	0.631	0.934	0.745	0.176	**0.020**	0.899	0.559
**CSF1PO**	0.766	0.193	0.808	0.443	0.342	0.088	0.707
**D5S818**	0.092	0.236	0.143	0.071	0.693	0.429	0.146
**D13S317**	0.085	0.547	0.629	0.767	0.959	0.847	0.420
**D7S820**	0.545	**0.010**	**0.000**	0.112	**0.002**	0.211	0.074
							
**Locus**	Serbia vs Kosovo (Albanians)	Serbia vs Greece	Serbia vs Slovenia	Croatia vs North Macedonia	Croatia vs Hungary	Croatia vs Bosnia and Herzegovina	Croatia vs Romania
							
**D3S1358**	0.058	0.819	0.140	0.754	0.267	0.714	0.762
**VWA**	0.945	0.465	0.316	0.573	0.085	0.910	0.578
**FGA**	0.517	0.633	**0.007**	0.703	0.718	0.213	0.098
**TH01**	**0.022**	0.075	0.091	**0.008**	0.167	0.536	0.000
**TPOX**	**0.004**	0.076	0.350	0.166	**0.013**	0.878	0.506
**CSF1PO**	**0.001**	0.969	0.224	0.334	0.183	0.084	0.634
**D5S818**	0.914	0.573	0.555	**0.012**	0.691	0.439	0.027
**D13S317**	0.280	**0.036**	0.555	0.678	0.808	0.386	0.590
**D7S820**	**0.003**	**0.004**	**0.019**	0.342	0.243	0.224	0.010
							
**Locus**	Croatia vs Kosovo (Albanians)	Croatia vs Greece	Croatia vs Slovenia	North Macedonia vs Hungary	North Macedonia vs Bosnia and Herzegovina	North Macedonia vs Romania	North Macedonia vs Kosovo (Albanians)
							
**D3S1358**	0.137	0.112	0.504	0.364	0.754	0.899	0.371
**VWA**	0.622	0.060	0.133	0.247	0.810	0.773	0.593
**FGA**	0.165	**0.014**	**0.042**	0.390	0.506	0.595	0.644
**TH01**	**0.000**	**0.000**	0.725	**0.044**	**0.034**	0.716	0.780
**TPOX**	**0.002**	0.057	0.575	**0.005**	0.230	0.655	0.398
**CSF1PO**	**0.000**	0.768	0.237	0.201	0.514	0.525	0.120
**D5S818**	0.293	**0.021**	0.196	0.083	**0.016**	0.113	0.202
**D13S317**	0.456	**0.001**	0.138	0.841	0.662	0.360	0.435
**D7S820**	**0.000**	**0.001**	0.604	0.839	0.645	0.823	0.007
**Locus**	North Macedonia vs Greece	North Macedonia vs Slovenia	Hungary vs Bosnia and Herzegovina	Hungary vs Romania	Hungary vs Kosovo (Albanians)	Hungary vs Greece	Hungary vs Slovenia
**D3S1358**	0.459	0.308	0.629	0.179	0.297	0.932	0.228
**VWA**	0.436	0.623	0.867	0.264	0.939	0.973	0.054
**FGA**	0.266	0.266	0.281	0.065	0.110	**0.011**	0.097
**TH01**	0.971	**0.018**	0.534	**0.014**	**0.001**	**0.006**	0.370
**TPOX**	0.089	0.249	0.395	**0.010**	**0.000**	0.251	0.215
**CSF1PO**	0.577	0.669	0.314	0.810	**0.000**	0.206	0.097
**D5S818**	0.131	0.426	0.457	0.505	0.834	0.293	0.420
**D13S317**	0.823	0.715	0.831	0.651	0.258	0.094	0.510
**D7S820**	0.654	0.205	0.238	0.622	**0.002**	0.776	0.141
**Locus**	Bosnia and Herzegovina vs Romania	Bosnia and Herzegovina vs Albanians (Kosovo)	Bosnia and Herzegovina vs Greece	Bosnia and Herzegovina vs Slovenia	Romania vs Kosovo (Albanians)	Romania vs Greece	Romania vs Slovenia
**D3S1358**	0.888	0.235	0.766	0.439	0.124	0.203	0.417
**VWA**	0.676	0.981	0.841	0.256	0.670	0.373	0.806
**FGA**	0.490	0.670	0.109	0.257	0.352	0.301	0.127
**TH01**	**0.024**	**0.001**	**0.019**	0.880	0.527	0.749	0.016
**TPOX**	0.550	**0.019**	0.581	0.682	0.079	0.131	0.313
**CSF1PO**	0.393	**0.017**	0.088	0.236	**0.000**	0.582	0.290
**D5S818**	0.091	0.431	0.142	0.155	0.719	0.102	0.196
**D13S317**	0.468	0.103	0.317	0.968	0.458	**0.004**	0.104
**D7S820**	0.378	**0.000**	0.078	0.472	**0.010**	0.798	0.073
**Locus**	Kosovo (Albanians) vs Greece	Kosovo (Albanians) vs Slovenia	Greece vs Slovenia
**D3S1358**	0.137	0.288	0.096
**VWA**	0.918	0.280	0.122
**FGA**	0.098	0.139	**0.000**
**TH01**	0.555	**0.002**	**0.005**
**TPOX**	**0.000**	**0.010**	0.377
**CSF1PO**	**0.005**	0.181	0.281
**D5S818**	0.741	0.602	0.897
**D13S317**	**0.017**	**0.064**	0.734
**D7S820**	**0.007**	**0.001**	**0.012**

**Figure 3:**
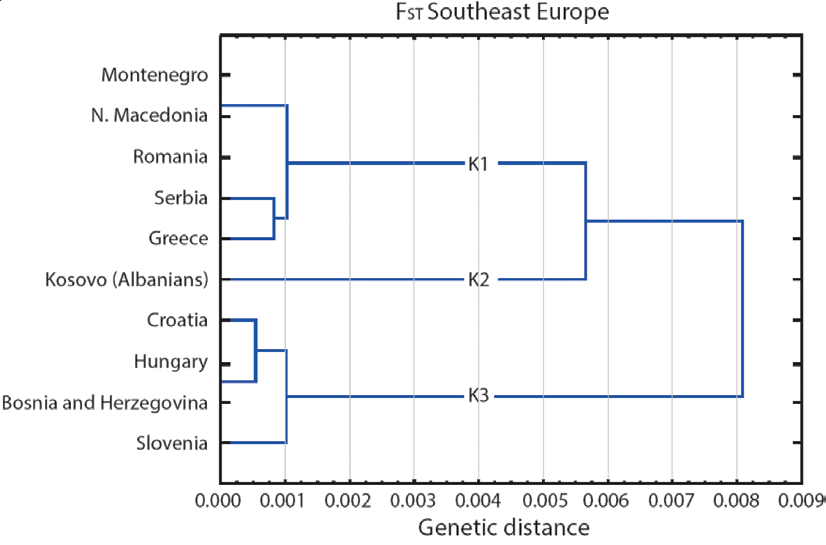
Clustering dendrogram obtained by Ward's method (Ward et al. 1963) based on genetic distances (F_ST_) between 10 populations of Southeast Europe with clusters K1, K2, and K3.

The grouping results for the populations of Southeast Europe based on F_ST_ genetic distances obtained by the Ward method are presented as a dendrogram (Figure 3). Three main clusters are visible. The first consists of the populations of Montenegro, Macedonia, Romania, Serbia, and Greece. The second cluster encompasses only the population of Albanians from Kosovo, and the third cluster consists of the populations of Hungary, Bosnia and Herzegovina, Croatia, and Slovenia. The greatest genetic distance was observed between the populations of Albanians from Kosovo and the Hungarian population.

### Structure assessment of Croatian subpopulations

**Figure 4:**
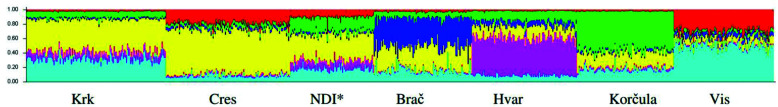
The population structure analysis of studied islands (n = 733) obtained by using the Structure 2.3.3 program and assuming an increasing number (3 to 7) of discriminating clusters. Each individual is represented by a vertical line consisting of K segments (each K segment is represented by a different color). The length of the segments is proportional to the estimated proportion in the statistically determined genetic group. Of all 10 runs, the ones with the highest value of ln (PD) for K = 3, K = 4, K = 5, K = 6, and K = 7 are shown graphically. *North Dalmatian islands: Ugljan, Pašman, Dugi otok.

Population genetic structure at the individual level was additionally estimated with the Structure program (34). A sample of exclusively island populations was used, since a preliminary study including the mainland sample determined the most reliable value for K = 1. In order to determine the most reliable K, the obtained results were processed with Structure Harvester. ΔK values were obtained, which more accurately estimate the value of K. A strong signal for K = 6 is visible in Figure 4, suggesting that the previously defined 7 island populations were grouped into 6 separate populations. Based on the determined most reliable K value (K = 6), the optimal presentation was 6 genetically different founder populations. Krk and NDI grouped together, and were separated from other populations at K = 4 and above. Furthermore, the separation of all other populations is visible. Cres and Vis were already separated at K = 3, while Brač and Hvar were separated at K = 5. The population of the island of Korčula was also separated from all others already at K = 5.

### Structure assessment of Southeastern European populations

**Figure 5:**
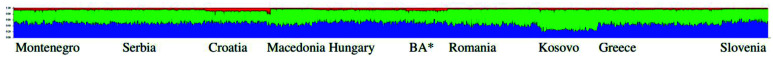
The population structure analysis of the studied Southeast European populations previously defined as 10 populations and presented by the Structure 2.3.3 program assuming an increasing number (3 to 7) of discriminating clusters. Each individual is represented by a vertical line consisting of K segments (each K segment is represented by a different color), and the length of these segments is proportional to the estimated share in the statistically determined genetic group. Since the sample size disparity may affect the determination of population structure (40), the total number of samples for Croatian population (n = 1230) was reduced (n = 158). Samples were randomly selected. *B&H – Bosnia and Herzegovina

The posterior probability (lnP (D)) obtained using the Structure program was highest for K = 1 and gradually decreased for each subsequent K. According to the second criterion for determining the most reliable K (40), taking into account ΔK, a strong signal was evident for K = 3, which would indicate the division of the Southeastern European sample into three groups. Population structure analysis of Southeastern European populations is presented for K = 3 to K = 7 (Figure 5). Based on the determined most reliable K value (K = 3), the optimal presentation was three genetically different founder populations. Thus, at K = 3, the population of Albanians from Kosovo stands out, and, to a lesser extent, the population of Croatia, Bosnia and Herzegovina, and Hungary.

## Discussion

### Genetic structure analysis

The total genetic differentiation coefficient (F_ST_) among all analyzed subpopulations of Croatia was low and amounted to 0.005, which means that only 0.5% of genetic diversity was influenced by differences between subpopulations. Since these geographically close populations share a common evolutionary history but have biological and socio-cultural specifics that have differently shaped their genetic structure, this study investigated how these populations related to each other and what the degree of their isolation was.

When looking at individual pairs of populations, 86% of population pairs were genetically different from each other as much as any two randomly selected European populations. Namely, among the largest European countries, the conservative upper limit of F_ST_ values was 1% according to the National Research Council (NRC) (41). This was significantly above the F_ST_ value of 0.0028, ie, 0.28% obtained based on STR marker analysis in 11 different European countries (42). However, according to the NRC, the F_ST_ value for isolated populations was 0.03, ie, 3%. Taking into account the recommended limit for isolated populations, the F_ST_ values of all analyzed island population pairs in this study were much lower than stated, and therefore the limit of 0.01 (1%) would be more appropriate.

Out of a total of 28 population pairs, no difference was found for two Croatian population pairs (mainland-Brač and Krk-NDI) at any of the analyzed loci. This finding might be explained by the fact that Brač and Krk are close to the mainland with good transportation connections and therefore less isolated than outer islands. Of the remaining population pairs, 14% differed in a slightly higher percentage. Thus, the highest degree of genetic differentiation was observed between Hvar and Vis (1.6%), which indicates an extremely high genetic diversity of these two populations. The remaining three population pairs with F_ST_ values >1% were Cres and Vis, Korčula and Vis, and Cres and Hvar, which also indicates the genetic diversity of these population pairs. On the other hand, allelic frequencies on the island of Brač and mainland, and Krk and NDI, which according to historical demographic data were founded by genetically similar ancestors, did not differ significantly. Previous studies of Eastern Adriatic islands observed a high degree of diversity (F_ST_) among most of the investigated population pairs. The diversity within the islands, ie, their settlements (subpopulations), has, for example, been established on Hvar, Krk, Brač, and Korčula (2,15). The greatest genetic similarity in this study was observed between the populations of mainland, Brač, Krk, and NDI, while Vis, as one of the remotest inhabited island in the Adriatic, was most different from all other studied subpopulations.

Genetic differentiation of Southeastern European populations was also assessed with the F_ST_ index. Due to the reduced impact of endogamy at a higher level of population grouping, a lower coefficient of genetic diversity was found among the populations of Southeastern Europe – only 0.16%, which indicates a homogeneous distribution of alleles of the studied loci at the level of “general” populations of Southeast Europe. Similar F_ST_ values (0.28%) were found among 11 European countries in an analysis based on microsatellite markers (42). The reason for this finding could be the socio-cultural origin of these populations in comparison with other studied European populations. Namely, they are the descendants of Illyrians, the autochthonous population of this area, which gradually intermixed with Romans, Slavs, and other more recent newcomers in the history of this area (43).

### Structure of island subpopulations according to the Bayesian approach

The genetic population structure at the individual level was also estimated by using the Structure software (34). A preliminary analysis, which included all Croatian subpopulations, determined the most reliable value of K = 1, ie, did not find a structure within the sample. Similar results were presented previously by Martinović Klarić et al (9). Given that human populations generally show a low degree of genetic differentiation, this result was expected. Namely, inter-population differences in Europe are very low (F_ST_ = 0.7%) (39), despite the presence of specific populations such as the Basques or the population of Sardinia. Due to the established low level of genetic differentiation (F_ST_) between defined (ancestral) populations of Croatia (from 0.001 to 0.016), the “LocPrior” model was used (36).

Furthermore, the mainland population was represented by a significantly larger number of participants (N = 497) than island populations (N = 82-137). Since disproportionate sample sizes may affect the determination of population structure (39), we hypothesized that isolating the mainland population from the sample will make it easier to find structure among the islands. The results of the analysis of 9 STR loci conducted in this study showed that even such a small number of loci with high heterozygosity is sufficient to determine structural division in case of population differentiation. Namely, in small isolated populations allelic frequencies can become significantly different from the founding population in a very short period of time due to genetic drift (44). The populations of the island of Krk and NDI exhibited a small genetic distance and can be considered as one population. The most pronounced separation was shown for the island of Vis, followed by the island of Cres, which is consistent with their separation into separate clusters based on genetic distances.

The established and most reliable value of K = 6 shows that these seven pre-defined island populations are grouped into six predicted, genetically different founder populations. This speaks in favor of the continued isolation of the eastern Adriatic Island communities and their mutual genetic diversity, and the existence of weak, but existing sub-structuring at the Croatian population level.

### Population structure of Southeastern Europe according to the Bayesian approach

The analysis showed greater homogeneity of the populations of this hierarchical group. Namely, while island subpopulations were separated into six predicted genetically different founder populations (K = 6), populations of Southeastern Europe split into only three different founder populations (K = 3). Due to the reduced influence of endogamy, lower genetic differentiation was found in Southeastern Europe than at the level of the Croatian subpopulations. To a lesser extent, the segregation of the populations of Croatia and Bosnia and Herzegovina and Hungary was visible, which is in line with their grouping into a common cluster based on F_ST_ analysis. This finding is not surprising, since they are geographically neighboring populations.

The isolation of the Albanian population from Kosovo confirms the results of previous analyses (27), where the largest genetic distance was determined for this population, as well as its separation into a special cluster. Albanians are non-Slavic speakers in the Western Balkans region. They are believed to be descendants of Illyrians with different cultural, demographic, and linguistic history compared with the neighboring populations of Slavic origin. Despite their widespread migration all over the European continent, traditional social-grouping of Albanians still remains strong, which may explain long-term genetic isolation (45,46).

This meta-analysis provides a systematic overview of the genetic sub-structuring in Croatia and in a wider Southeast-European context. It also highlights the importance of isolated island population in the making of a population’s genetic landscape. There are certain limitations to this study. A meta-analysis includes data from many different sources, which has certain disadvantages. Among others, the number of STRs included in the study had to be reduced in order to enable comparisons. However, even with a limited number of STRs (only nine) a sub-structure was detected.

### Conclusion

The total genetic differentiation coefficient of Croatian subpopulations calculated by the F_ST_ method was higher at the level of the Croatian population (0.005) than at the level of Southeast Europe (0.002). Namely, the assessment of the genetic population structure for Croatia defined 6, and for the population of Southeastern Europe 3 genetically different clusters. In the population of Croatia, the subpopulation of the island of Vis showed the most pronounced separation, and in the population of Southeastern Europe the population of Albanians from Kosovo, followed by the populations of Croatia, Bosnia and Herzegovina, and Hungary. The established higher structure of Croatian subpopulations in relation to Southeastern Europe suggests the existence of a certain degree of genetic isolation, most likely due to the influence of endogamy within rural island populations.
